# Prognosis, Biology, and Targeting of *TP53* Dysregulation in Multiple Myeloma

**DOI:** 10.3390/cells9020287

**Published:** 2020-01-24

**Authors:** Erin Flynt, Kamlesh Bisht, Vinidhra Sridharan, María Ortiz, Fadi Towfic, Anjan Thakurta

**Affiliations:** 1 Celgene Corporation, Summit, NJ 07901, USA; eflynt@celgene.com (E.F.); kbisht@celgene.com (K.B.); vsridharan@celgene.com (V.S.); 2 Celgene Corporation, Celgene Institute for Translational Research Europe, 41092 Sevilla, Spain; mortiz@celgene.com; 3 Celgene Corporation, San Diego, CA 92121, USA; ftowfic@celgene.com

**Keywords:** P53, myeloma, del17p, genomics, high-risk

## Abstract

Multiple myeloma (MM) is the second most common hematological cancer and is characterized by genetic features including translocations, chromosomal copy number aberrations, and mutations in key oncogene and tumor suppressor genes. Dysregulation of the tumor suppressor *TP53* is important in the pathogenesis of many cancers, including MM. In newly-diagnosed MM patients, *TP53* dysregulation occurs in three subsets: monoallelic deletion as part of deletion of chromosome 17p (del17p) (~8%), monoallelic mutations (~6%), and biallelic inactivation (~4%). Del17p is an established high-risk feature in MM and is included in current disease staging criteria. Biallelic inactivation and mutation have also been reported in MM patients but are not yet included in disease staging criteria for high-risk disease. Emerging clinical and genomics data suggest that the biology of high-risk disease is complex, and so far, traditional drug development efforts to target dysregulated *TP53* have not been successful. Here we review the *TP53* dysregulation literature in cancer and in MM, including the three segments of *TP53* dysregulation observed in MM patients. We propose a reverse translational approach to identify novel targets and disease drivers from *TP53* dysregulated patients to address the unmet medical need in this setting.

## 1. Introduction

Multiple myeloma (MM) is a malignancy of fully differentiated B cells and represents the second largest hematological cancer in the US [1]. Over the last two decades, remarkable progress in the development of therapeutics has resulted in approval of novel therapies that include immunomodulatory drugs, proteasome inhibitors, and more recently an anti-CD38 antibody, with significant increases in both progression free and overall survival of patients [2,3]. However, clinical benefit is not uniform, and the disease remains incurable. Patients with high-risk disease are one segment that are underserved by current therapies [3]. The Revised ISS (R-ISS) criteria that is used to risk stratify MM patients at diagnosis includes select cytogenetic abnormalities (CA) to define high-risk MM: the presence of one or more of: 4;14 or 14;16 translocations, or deletion of chromosome 17p (del17p) [4]. While del17p, which includes the *TP53* gene, is a known high-risk marker in MM, variability in cytogenetic assay cutoff has resulted in a heterogenous population of patients with this abnormality being designated as high-risk. The Myeloma Genome Project (MGP) has identified high-risk patients using molecular methods to circumvent challenges associated with traditional methods. MGP identified two high-risk patient segments that included *TP53* aberrations: Double Hit MM (DHMM) which includes patients with biallelic inactivation of *TP53* (a deletion and a mutation) and a second segment of patients harboring del17p in a high cancer cell fraction (CCF) [5,6]. In this review, we discuss the current understanding of P53 in cancer, and the prognosis and biology of patients harboring distinct abnormalities involving *TP53*: (1) del17p, (2) mutations, and (3) biallelic inactivating events. These *TP53* aberrations can be present in newly diagnosed MM (NDMM) but may also be acquired in later stages of the disease following treatment. Emerging data and our ongoing analyses suggest a complex molecular basis of P53 dysregulated high-risk MM. Here, we review *TP53* aberrations in cancer, in MM including clinical prognosis in MM, the biology of P53 inactivation, and attempts to target *TP53* in drug development. We suggest a path forward for developing new therapies by taking a reverse translational approach to address the unmet need for these patients. 

## 2. P53 Aberrations in Solid Tumors and Hematological Malignancies

*TP53* was originally discovered as a binding partner of simian virus 40 large T antigen in virally- transformed cells [7,8,9,10]. Initially it was classified as an oncogene, but later work established its role as a tumor suppressor [11]. A variety of inactivating *TP53* mutations have been reported in human cancers and germline mutations in *TP53* are a hallmark of Li-Fraumeni syndrome, a hereditary cancer predisposition disorder [11,12,13]. 

Approximately 50% of human cancers have *TP53* alterations [14,15,16]. In The Cancer Genome Atlas (TCGA) dataset that includes 32 distinct studies and over 10,000 cancer cases, the prevalence of *TP53* mutations are 15.20%, deletions 15.90%, and biallelic inactivation events are 22.02% of cases [17]. In this dataset, ovarian serous cystadenocarcinoma, uterine, and lung cancers have the highest prevalence of *TP53* abnormalities (~90% of cases) while paraganglioma had the fewest at only 0.50% [18]. Other groups have also reported high prevalence of *TP53* abnormalities in solid tumors, particularly ovarian, pancreatic, breast, and small cell lung cancer [13,19,20,21]. However, Li and colleagues analyzed data from 7893 patients and found that *TP53* mutations were only prognostically relevant in 9 cancer types in the TCGA dataset including lung adenocarcinoma, hepatocellular carcinoma, head and neck squamous cell carcinoma, acute myeloid leukemia (AML) and clear cell renal carcinoma [18]. Approximately 80% of *TP53* mutations are missense mutations and are localized in the DNA-binding domain. Eight of these mutations (R175, V157F, Y220C, G245, R248, R249, R273 and R282) account for ~28% of total mutations in *TP53* with R5, R248 and R273 being reported in multiple tumor types, suggesting that there is a selection for these mutant alleles in cancer [22]. 

In addition to single-allele missense mutations, loss of heterozygosity (LOH) in the second allele of *TP53* has been reported in multiple solid tumor studies and mutations in this allele were significantly higher (25–37%) than in non-del17P cases [12,15,23,24,25,26]. Analysis of *TP53* gene and pathway alterations in 32 tumor types from the TCGA dataset revealed that ~91% of cancers exhibit biallelic inactivation of the *TP53* gene. The second allele loss was due to either mutation, chromosomal deletion, or by copy neutral LOH [27]. Gene expression profiling of both cell lines and patient samples suggested that even monoallelic deletion of *TP53* can result in significantly lower expression levels [26,28]. 

**Figure 1:**
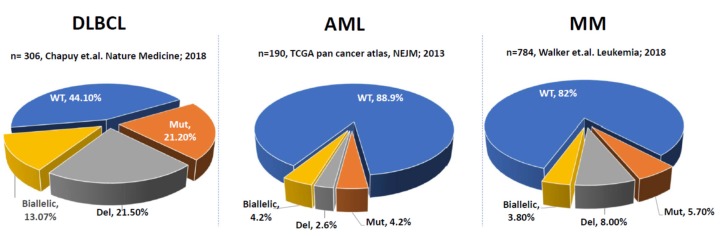
Prevalence of wild-type (WT), deletion (del), mutation (mut), and biallelic inactivation of *TP53* in select hematologic malignancies, DLBCL (**left**), AML (**middle**), and MM (**right**).

Compared to solid tumors, dysregulation of *TP53* is less frequent in hematological malignancies, for example, in diffuse large B-cell lymphoma (DLBCL) and AML, ~10–50% of cases have alterations in *TP53* (Figure 1). In DLBCL, biallelic inactivation is the most common *TP53* aberration (13%) while deletion and mutation are each present in ~20% of cases. In AML, alterations in *TP53* are less common with biallelic inactivation and mutation present in ~4% of cases each and deletion reported in only ~3% of cases. There is only one dataset with SNV data from 211 MM patients available in TCGA which lacks copy number variation (CNV) data, thus providing incomplete information about monoallelic versus biallelic inactivation of *TP53* in MM [29]. Our analysis from MGP demonstrated that deletion is the most common abnormality at 8%, followed by mutation (~6%) and biallelic inactivation (~4%) (Figure 1). Even though the prevalence of *TP53* aberrations is high across multiple tumors, their biological effects are still poorly understood. Various studies have suggested that missense mutations in *TP53* are gain of function (GOF) mutations and confer oncogenic functions to P53 [22,30,31,32,33,34,35,36,37]. In contrast, other studies suggest that missense mutations in *TP53* are loss of function (LOF) and act through a dominant-negative mechanism affecting oligomerization [38,39,40,41,42]. A recent report conducted a detailed analysis of *TP53* hot spot missense mutations in human myeloid malignancies applying genome editing, saturation mutagenesis screening and mouse models [40]. This analysis showed that in AML, *TP53* mutations do not confer a neomorphic GOF potential but instead, are dominant negative in nature and affect the tumor suppressor function of the protein [40].

## 3. Prognosis of Del17p/*TP53* Inactivation in Multiple Myeloma

### 3.1. Deletion of 17p in MM

Chromosomal aberrations, including translocations and CNVs are common in MM. However, heterogeneity in patient populations, detection methods, threshold (defined as the percentage of tumor cells positive, also known as CCF), sample sizes, and treatment regimens have made it challenging to interpret their impact on clinical outcome. The prognostic relevance of del17p was described in an analysis of NDMM patients in the Intergroupe Francophone de Myélome 99 (IFM99) trial [43]. Del17p (identified by fluorescent in situ hybridization [FISH]) in CD138-positive tumor cells was present in 11% of patients (58 out of 532) where patients with the deletion had significantly shorter median event-free survival (EFS; 14.6 vs. 35 mo, *p* < 0.001) and median overall survival (OS, 22.4 mo vs. not reached [NR], *p* < 0.001). 

Del17p was one of two markers that retained prognostic value for both EFS and OS in a multivariate analysis of a large NDMM patient dataset, and is included in the R-ISS as a standard part of risk assessment in the clinic [4]. However, one source of variability among studies has been the threshold used to determine whether patients are considered positive for del17p. In the IFM99 study, del17p patients had a median of 75% plasma cells positive for the deletion, and those with ≥60% positive cells (CCF >0.6) were considered high-risk. The majority of studies have used a cutoff of >20% to determine a significant impact on clinical outcome and some have shown that a patient should have ~60% cells positive for the deletion in order to be included in the high-risk del17p subgroup [25,43,44,45,46,47,48,49,50]. However, some groups have used thresholds below 20%, including one Phase 3 study that counted a patient positive for del17p if even a single cell was positive by FISH [51,52]. The use of different thresholds/CCFs, different size datasets, as well as different treatment regimens have resulted in discordance in the reported prognosis of del17p patients (Table 1). In NDMM, the median progression free survival (PFS) for patients with del17p has been reported to be approximately 15 months in several studies, with a range from 4 to 26 months (Table 1). Differences in threshold/CCF as well as treatment intensity impact the clinical outcomes of these patients. The study by Chang and colleagues, and that by An and colleagues reported shorter median PFS compared to others in Table 1, but it should be noted that these patients were treated with high-dose chemotherapy or chemotherapy plus either thalidomide or bortezomib.

The MGP systematically evaluated large FISH and genomic datasets for the association of CCF to OS in NDMM patients [6]. This analysis indicated that patients with greater than 55% del17p-positive cells (CCF > 0.55), had poor clinical outcomes, where patients had median PFS and median OS of only 14 and 36 months as compared to the low CCF group (≤0.55) who had median PFS of 24 months and median OS of 84 months (FISH dataset, *n* = 605) [6]. The CCF > 0.55 threshold to identify high-risk del17p patients (high-risk [HR] del17p, ~7% of NDMM) was validated in a meta-analysis across three datasets, the original test dataset from the IFM, as well as an independent FISH replication dataset (*n* = 235) and a genomic dataset (*n* = 108) [6].

The poor outcome of high-CCF del17p was also demonstrated in analysis of multiplex ligation-dependent probe amplification (MLPA) data, a multiplex polymerase chain reaction (PCR) method for simultaneously detecting CNVs at different genomic regions, where patients with MLPA cutoffs corresponding to ≥50% tumor cells positive for del17p had significantly shorter PFS and OS than those with fewer del17p-postive cells [47,53]. These data are consistent with earlier reports suggesting that a CCF of ~60% should be used to identify high-risk patients [43]. 

Several studies investigated clinical outcomes of relapsed and/or refractory MM (RRMM) patients with del17p (Table 2). Lakshman and colleagues reported data in 76 MM patients who were negative for del17p at diagnosis who later tested positive for del17p. The presence of del17p was detected at a median of 35.6 months after diagnosis (median of 2 lines of therapy), and was associated with short median PFS (30.1 vs. 23 mo; *p* = 0.032) and median OS (106.1 vs. 68.2 mo; *p* < 0.001) in comparison to patients without detectable del17p in the same timeframe [44,54]. Other reports of clinical outcomes in del17p-positive patients demonstrate the association of poor clinical outcome with median PFS ranging from 3.4 to 21.4 months, presumably impacted by threshold/CCF used as well as treatment intensity in individual patient cohorts. The recent analysis from Avet-Loiseau and colleagues demonstrates the impact of treatment intensity where patients with >60% del17p-positive cells had a median PFS of 15.47 months in the triplet arm versus only 5.1 months in the doublet arm [55]. Taken together these data demonstrate that patients with del17p have poor clinical outcomes, and that patients with high CCF del17p are at particularly high risk of early progression and death compared to low-CCF patients in both NDMM and RRMM settings. These findings are clinically relevant and CCF should be considered for evaluation of del17p in MM patients and integrated into risk stratification guidelines.

### 3.2. Biallelic Inactivation of TP53 in MM

Early reports from analyses of small numbers of patients suggested an association between deletion on one allele and mutation on the second allele of chromosome 17p, putatively resulting in complete inactivation of P53 function [25,44,46]. These studies reported clinical outcome of patients with biallelic inactivation as well as del17p alone; however, small numbers limited the ability to differentiate the impact of biallelic versus monoallelic inactivation. In a cohort of 92 NDMM patients, Lodé and colleagues reported that among 54 patients with del17p, 20 (37%) also had a mutation in *TP53*; however, no differences in survival were observed between patients with complete inactivation of *TP53* and those with del17p alone [25]. In a larger NDMM dataset (*n* = 779) where both *TP53* mutation and FISH data were available (*n* = 72), a significant correlation between mutation and deletion was observed [44]. Patients with biallelic inactivation (*n* = 7) had significantly shorter PFS and OS than patients with monoallelic inactivation (*n* = 26, del17p or *TP53* mutation alone) where 3-year OS was 29% versus 84% and 3-year PFS was 29% versus 73%. This study did not separately report the outcomes for patients with only a mutation or deletion.

From the MGP, using the largest uniformly-processed genomic dataset from NDMM patients (*n* = 1273), DHMM was identified and independently validated as a molecularly-defined high-risk group. DHMM is ~6–8% of NDMM and includes two groups of patients, (a) biallelic inactivation of *TP53* and (b) amplification (≥4 copies) of 1q21 with ISS3 with median PFS of 15.4 months and median OS of 20.7 months [5,62]. Patients with biallelic inactivation of *TP53* had significantly shorter PFS than patients with monoallelic del17p, (18 mo estimate: PFS 36% vs. 76%; OS: 58% vs. 90%) [5]. However, this analysis did not consider the CCF >0.55 cut off for determination of HR del17p.

**Figure 2:**
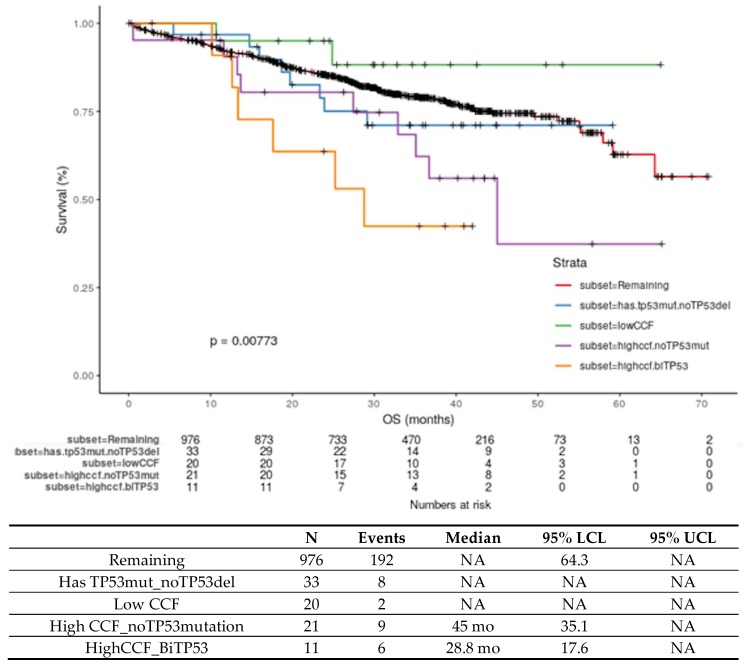
Kaplan-Meier survival curve for updated OS in months from the MGP dataset (*n* = 1061) showing patients with (a) CCF > 0.55 + mutation of *TP53* (highccf.biTP53, orange line), (b) CCF > 0.55 without a mutation in *TP53* (highccf.noTP53mut, purple line), (c) CCF < 0.55 (lowCCF, green line), (d) *TP53* mutation without a deletion of 17p (has.TP53mut.noTP53del, blue line), and remaining patients who did not fall into any of these groups (red line). The number in each group, number of events, median OS, and lower and upper 95% lower/upper confidence intervals (LCL, UCL) are provided in the table.

Using the >0.55 CCF cutoff defined by genomics-based methods, analysis from MGP identified high-risk del17p patients as discussed in Section 3.1. The MGP data also showed a significant association between the presence of CCF >0.55 and of mutation on the second allele of *TP53*, where 27 of 28 patients with a *TP53* mutation had CCF >0.55 [6]. Patients with biallelic inactivation had significantly shorter PFS than patients with one wild-type copy of *TP53* [6]. In an updated analysis of this dataset with longer clinical follow-up, patients with HR del17p + *TP53* mutation have the shortest OS (Figure 2, orange line, median 28.8 mo, *p* = 0.008). Figure 2 provides updated OS data for patients with HR del17p (median 45 mo) in the MGP dataset. These data highlight that NDMM patients with either biallelic inactivation of *TP53* or high CCF del17p have very poor clinical outcome. Together, these two groups of P53 dysregulated patients comprise approximately 6–7% of the NDMM population (the addition of the DHMM amp1q segment would bring this to ~10% of NDMM).

The timing and order of acquisition of deletion and mutations leading to biallelic inactivation of *TP53* in MM patient samples has not been widely studied. A recent preclinical study was performed in isogenic AMO-1 MM cell lines containing monoallelic and biallelic *TP53* variants followed by *in-vitro* competition assays. Interestingly the authors found that biallelic *TP53* inactivated cells outcompete monoallelic *TP53* variant containing cells. Although this study was limited to one cell line, the data provided some experimental evidence for the acquired proliferation fitness of biallelic clones [63]. Due to the recessive nature of tumor suppressor mutations, biallelic inactivation is generally a prerequisite for acquiring an oncogenic or tumor maintenance phenotype. While biallelic inactivation would result in loss of P53 function, it is unclear what drives the high-risk feature of a higher clonal fraction of del17p cells with respect to P53 function.

### 3.3. Monoallelic Mutation of TP53 in MM

**Figure 3:**
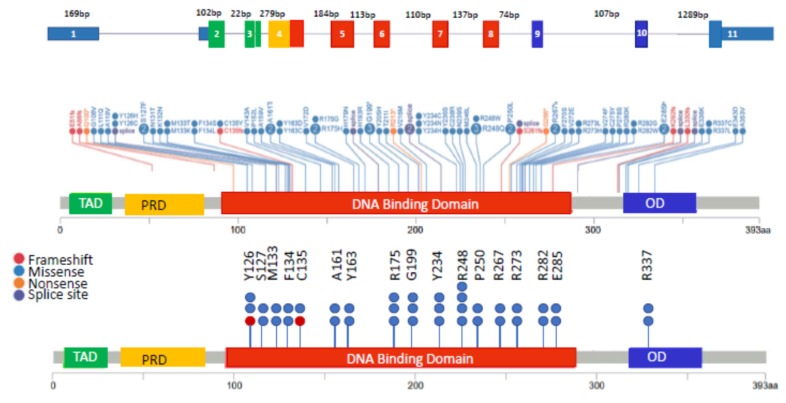
*TP53* mutation spectrum in MM: Top: Structure of *TP53* gene with numbered exons, colors correspond to domains highlighted in middle and bottom panels. Middle: *TP53* mutations in MM by type and location within the gene. Bottom: Hotspot mutations in *TP53* in MM. Only mutation sites that were detected in ≥2 patients in the MGP dataset (*n* = 863) are shown. Each circle represents one patient with blue representing missense mutations, purple representing splice site mutations, red representing frameshifts, and orange representing nonsense mutations. TAD = Transactivation domain TAD (green), PRD = proline rich domain (yellow), OD = oligomerization domain (blue).

In NDMM, *TP53* mutations are generally present in 3–8% of patients (Figure 1) [5,46,64]. As in other tumor types, *TP53* mutations in MM are spread across the entire gene, with many mutations occurring within the DNA-binding domain [5]. Figure 3 highlights the structure of the *TP53* gene (top), mutations in *TP53* in the MGP dataset (middle panel) as well as the sites most frequently mutated (Figure 3, bottom) in ≥2 patients. *TP53* mutation was identified as a driver event in MM [65]. However, the relevance of monoallelic *TP53* mutation as an independent poor prognostic marker in MM has not been established. Owen and colleagues concluded that *TP53* mutations were rare events in MM (detected in 1/31 [3%] patients) and were therefore of limited prognostic value [64]. Chng and colleagues reported the prognosis in NDMM where *TP53* mutations were detected in 3% (9/268) of patients and were associated with short OS compared to patients without a mutation, however, it should be noted that ~50% of this cohort of patients also had a del17p [46,66]. Another study reported that patients with *TP53* mutation (*n* = 20) did not have a significantly different outcome compared to those without a mutation [25]. In the MGP dataset, there were 33 patients with monoallelic mutation of *TP53*, and after a median follow-up of 29.8 months, the OS of these patients (Figure 2, monoallelic mutation, blue line) was not significantly different than patients without a *TP53* abnormality (Figure 2, remaining, red line). Longer follow-up or analysis of larger datasets are needed to clarify the prognostic value of monoallelic *TP53* mutation in MM patients. 

Nonetheless, *TP53* mutations could be important drivers for maintaining/propagating MM clones in association with co-occurring driver gene mutations. The variant allele frequencies of the *TP53* mutations are sub-clonal for the majority of the mutations observed in the 33 patients within the MGP dataset. The functional consequence of *TP53* mutations in MM is yet to be experimentally determined, but in silico predictions suggest that multiple hot spot mutations may have a damaging effect. Figure 3 (bottom) and Table 3 summarizes the *TP53* hot spot mutations (detected in ≥2 patients) in MM and their predicted functional impact and score from three different in silico methods: Mutation Assessor, Sorting Intolerant from Tolerant (SIFT), and Polymorphism Phenotyping V2 (PolyPhen2) [67,68,69]. The majority of *TP53* hotspot mutations are predicted to be medium impact by Mutation Assessor and the same mutations are predicted as deleterious and damaging by SIFT and PolyPhen2 algorithms. Mutation Assessor classifies mutations into 4 categories based on functional impact score (FIS) to estimate the probability of phenotypic consequence of the mutation. A FIS score ≤0.8 is classified as neutral impact, FIS score of 0.8–1.9 as low impact, FIS score of 1.9–3.5 as medium impact and >3.5 as high impact. Among the hot spot mutations observed in MM, mutations at Y126, R175, R248, R273, and R282 are also common in other tumor types in the TCGA database [17]. Laboratory validation is needed to confirm the predicted functional consequence of the observed mutations in MM cells. 

## 4. Biology of Del17p/P53 Inactivation 

In normal cells, P53 is maintained at a low level by a series of regulators and is activated by a variety of stress stimuli. P53 controls a vast genetic network, complex transcriptional programs, and diverse biological responses that have been recently reviewed [70]. The most studied function of P53 is its ability to promote cell cycle arrest and apoptosis via transcription of P21 in response to DNA damage, which is deemed central to its role in tumor suppression. P53 also controls many other biological processes including metabolism, proliferation, inflammation, autophagy and epithelial to mesenchymal transition [70]. The complexity of the P53 signaling network has made interpretation of P53′s function, and consequences of its dysfunction challenging—particularly when considering the impact of cell type and mechanism of inactivation (e.g., mutation, deletion, or both). Here we review the major roles of P53 and the impact of P53 dysfunction in cancer overall and in MM specifically.

### 4.1. Role of P53 in Genomic Instability, DNA Repair, Aneuploidy, and Checkpoint Control

P53 maintains genome stability by inducing cell cycle arrest, senescence, or apoptosis upon DNA damage to reduce the risk of propagation of a defective genome. Genome instability is an inherent characteristic of almost all human cancers and is accompanied by dysregulation of several cellular processes, such as DNA replication, G2/M cell cycle checkpoint control, chromosomal segregation, DNA repair, and genome integrity. P53 is frequently inactivated in cancer resulting in tumors characterized by gross structural defects, chromosomal missegregation, ploidy changes, and higher prevalence of chromothripsis [12,14,49,71,72,73,74]. A pan-cancer analysis demonstrated an association between aneuploidy and mutation of *TP53* [14]. One of the mechanisms by which dysregulated P53 is thought to contribute to aneuploidy is via regulation of G2/M processes and centrosome amplification [75,76,77]. Multiple studies have shown a complex link between proteins involved in cell cycle regulation, mitotic checkpoints, genomic instability, tumorigenesis, and the P53 pathway [78,79,80,81]. A tightly regulated feedback loop has been reported between P53 and mitotic kinases (eg, WEE1, PLK1, NEK2, BUB1, TTK, AURKB and PLK1) [79,82,83,84]. Phosphorylation of P53 in response to mitotic spindle damage has also been reported [85]. The intricate details of the mechanisms and regulatory signals between mitotic kinases and P53 remain poorly understood, but P53 dysfunction disrupts this critical regulation, resulting in abrogation of the G1 checkpoint and upregulation of mitotic kinases allowing cells to override the G2/M checkpoints and contribute to genome instability and tumorigenesis. A clear understanding of the biology of dysregulation of P53 dependent physiological processes in biallelic, del17p, and mutated P53 patients is critical in identifying new anti-cancer targets.

### 4.2. P53 Synthetic Lethality

**Figure 4:**
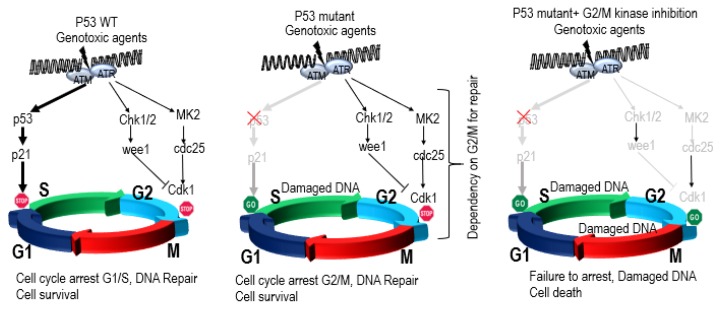
Mechanism of synthetic lethality of P53 with G2/M kinases: When cells are treated with genotoxic agents, they undergo a P53-mediated G1/S arrest to allow time for DNA repair, but in a P53-deficient background, the G1/S checkpoint is absent, and cells are dependent upon the G2/M checkpoint for survival.

**Figure 5:**
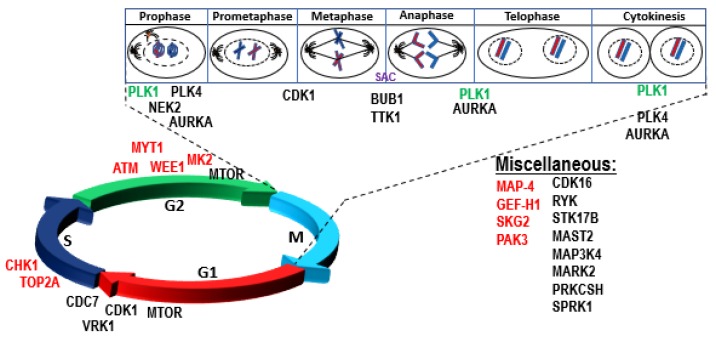
Synthetic lethal interaction of P53 with cell cycle and other genes and their distribution across cell cycle. Experimentally validated or detected in functional genomic screens (red), in silico predicted (black) and both (green). PLK1 = polo-kinase 1, ATM = ataxia telangiectasia mutated, GEF-H1 = guanine nucleotide exchange factor-H1, MK2 = MAP-kinase activated protein kinase 2, SGK2 = serine/threonine kinase, CHK1 = Serine/threonine-checkpoint kinase 1, MAP4 = microtubule-associated protein 4, MYT-1 = Membrane-associated tyrosine- and threonine-specific cdc2-inhibitory kinase, Wee1 = nuclear serine/threonine kinase 1, CDK16 = cyclin-dependent kinase 16, RYK = receptor-like tyrosine kinase, MTOR = mechanistic target of rapamycin, STK17B = serine/threonine kinase 17b, PLK4 = polo-like kinase 4, MAST2 = microtubule associated serine/threonine kinase 2, MAP3K = 4 mitogen-activated protein kinase kinase kinase 4, MARK2 = MAP/microtubule affinity-regulating kinase 2, CDK1 = cyclin-dependent kinase 1, NEK2 = NIMA (never in mitosis gene a)-related kinase 2, PRKCSH = protein kinase C substrate 80K-H, AURKA = aurora kinase A, BUB1 mitotic checkpoint serine/threonine kinase, CDC7 = cell division cycle 7 homolog, SRPK1 = SRSF protein kinase 1, TTK = TTK protein kinase, VRK1 = vaccinia related kinase 1.

One way to explore new targets is by identifying vulnerabilities of cancer cells with dysregulated P53 that could provide opportunities to selectively kill or inhibit the growth of P53-deficient cells versus those with wild-type P53 (Figure 4). Loss of the G1/S cell cycle checkpoint in P53-deficient cancer cells renders them entirely dependent on the G2/M checkpoints to maintain genome integrity [86]. Such dependency exposes a unique vulnerability of P53-deficient cancer cells, resulting in synthetic lethal relationship between P53 and multiple genes/pathways (Figure 5). P53-deficient cancer cells exhibit synthetic lethal interactions with ionizing radiation and genotoxic agents (e.g., cisplatin, camptothecin, doxorubicin). For example, P53-deficient cells are more sensitive to genotoxic stress when treated with inhibitors of ATR, Chk1, PLK1, and Wee1 kinases versus cells with functional P53 [87,88,89,90,91]. P53-deficient cells have also been reported to be dependent on the p38MAPK/MK2 pathway for survival following treatment with DNA-damaging agents. MK2 depletion in P53-deficient cells suppressed Cdc25A-mediated S phase arrest following cisplatin treatment and Cdc25B-mediated G2/M arrest following doxorubicin exposure, resulting in mitotic catastrophe and tumor regression in vivo [92]. Further, inhibition of ATM also exhibited synthetic lethality with topoisomerase inhibitors in a P53-deficient background [93] (Figure 4). P53 synthetic lethality has also been reported with SGK2, PAK3, CHK1, Wee1 and Myt1 in cervical cancer cell lines [94,95].

Additionally, Wang and Simon employed a computational method to predict genes with P53 synthetic lethality. Using publicly-available cell line and gene expression datasets, they identified 18 kinases with potential synthetic lethal interactions with P53, including PLK1, NEK2, BUB1, and AURKA [96]. Another study identified a similar set of potential P53 synthetic lethal genes by analyzing publicly-available data in 33 different human cancer types to identify 120 genes that were overexpressed in P53 deficient cells/tumors, including 19 genes that were common across tumor types [97]. A significant number of these putative P53 synthetic lethal genes are potentially druggable mitotic kinases (AURKA, BUB1, BUB1B, CDK1, MELK, NEK2, PLK1 and TTK), with 6 out of 8 kinases common across both studies [96,97]. 

**Figure 6:**
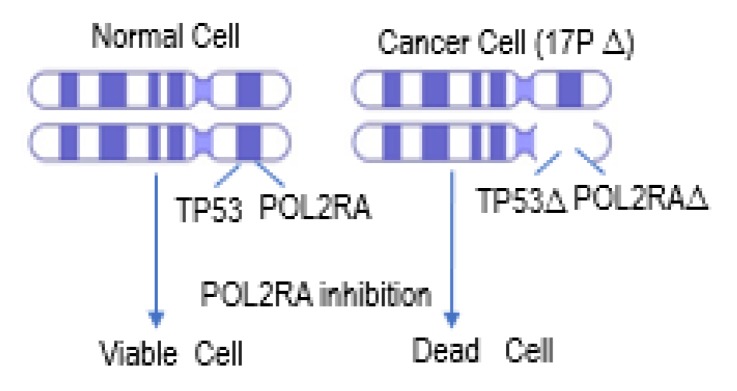
Example of P53 synthetic lethality with POLR2A (RNA polymerase II catalytic subunit), a co-deleted gene with deletion of chromosome 17p that results in vulnerability to POLR2A inhibition and synthetic lethality with α-amanitin.

The region of chromosome 17p that is commonly deleted includes several essential genes that are adjacent to *TP53*. For example, one of the largest subunits in the human RNA polymerase II complex (POLR2A) resides in close proximity to the *TP53* gene locus (Figure 6). Concomitant deletion of POLR2A with hemizygous *TP53* deletion has been reported in multiple human cancers [98,99]. Unlike *TP53* that is regulated post-transcriptionally and post-translationally, expression of POLR2A is directly correlated with gene copy number. Hence, the inhibition of the POLR2A gene in cells with hemizygous del17p genomic deletion resulted in synthetic lethality and increased cell death [99]. E3 ligase Ring-Box 1 (RBX1) has also been identified as another P53 synthetic lethal partner which regulates POLR2A-mediated mRNA synthesis by K63 linked ubiquitination. Inhibition of RBX1 in P53 deleted castration-resistant prostate cancer cell lines resulted in synergistic inhibition of cell growth [98]. 

#### P53 Synthetic Lethality in MM

MM has remarkable genomic instability that leads to accumulation of aberrations resulting in tumor progression, drug resistance, and metastasis [100]. For example, increased DNA double-stranded breaks (DSB) may lead to disease progression in MM [101]. Genomic instability provides a growth advantage and may allow acquisition of drug resistance, but it may also create vulnerabilities that can be exploited by targeting synthetic lethal interactions [102]. An example of this strategy is induced sensitivity of MM cells to poly (ADP-ribose) polymerase (PARP) inhibitors following 26S proteasome inhibition. Inhibition of the proteasome in MM cells abrogates H2AX polyubiquitination and abolishes recruitment of BRCA1 and RAD51 to DSB sites and homologous-recombination (HR)-mediated DNA repair [103]. Co-treatment of MM cells with proteasome inhibitors and PARP inhibitors leads to accumulation of unrepaired DNA DSBs and cell death [103]. Similarly, mutations in DNA editing enzymes, (eg, APOBECs) have been reported in a specific MM genomic subgroup that is associated with primary/secondary translocations, poor prognosis, drug resistance, oncogenic activation, and sub-clonal diversity [104,105,106,107,108,109,110]. APOBEC mutagenesis induces the DNA damage response and can result in cell death [111,112,113]. Therefore, loss of P53 enables tolerance to APOBEC-mutagenesis-induced DNA damage and promotes cancer cell survival [113]. Together, these data suggest that targeting genes with a synthetic lethal relationship with P53 could be an effective therapeutic approach for multiple P53-deficient malignancies. Additional research is needed to identify genes with significant P53 synthetic lethal relationships in MM. 

### 4.3. Biology of High-risk Del17p in MM 

The clinical relevance of del17p is well established in MM, but the exact mechanism by which del17p promotes aggressive disease biology remains unclear. Questions remain on the role of the deletion size, clonality, and cellular signaling. Without a better understanding of the features that are driving poor clinical outcome, development of effective therapies to target those features remains challenging. An integrative multi-omics analysis identified 12 distinct disease subsets of MM and indicated that del17p and DHMM, despite being associated with poor clinical outcome, did not all cluster together in one subset [114]. Instead, patients with DHMM or del17p were distributed across several subsets, including high-risk and non-high-risk groups indicating that these features are not the only drivers of the underlying high-risk biology.

The length of the deleted region can vary from a few mega bases (MBs) to deletion of the entire short arm of chromosome 17. The *TP53* gene is located in the minimally deleted region (0.25 MB) suggesting that it is a critical gene in the 17p13 region [50]. A deletion event usually involves several genes and it remains unclear how genes other than *TP53* contribute to tumorigenesis. A heterozygous deletion of a 4MB region in mouse chromosome 11B3, syntenic to human 17p13.1, showed that co-deletion of *TP53* along with Eif5a and Alox15b resulted in more aggressive disease [115]. Additional research is needed to improve our understanding of drivers of high-risk biology in MM patients with del17p.

## 5. Targeting TP53 in Drug Development

The majority of *TP53*-related drug development efforts have been directed towards designing therapies to exploit cancer-specific vulnerabilities associated with dysregulation of *TP53*, such as del17p, *TP53* mutations, *TP53* promoter methylation, and MDM2 overexpression [70,116]. The therapeutic utility of synthetic lethal interaction of P53 with POL2RA is currently being examined in preclinical models. The amanitin toxin, which inhibits POL2RA is being tested as an antibody-drug conjugate (ADC). In MM, anti-B cell maturation antigen (anti-BCMA) amanitin-ADCs have shown efficacy and tolerability in preclinical models [117,118]. Additional testing is needed to determine clinical efficacy and tolerability of these agents. 

**Figure 7:**
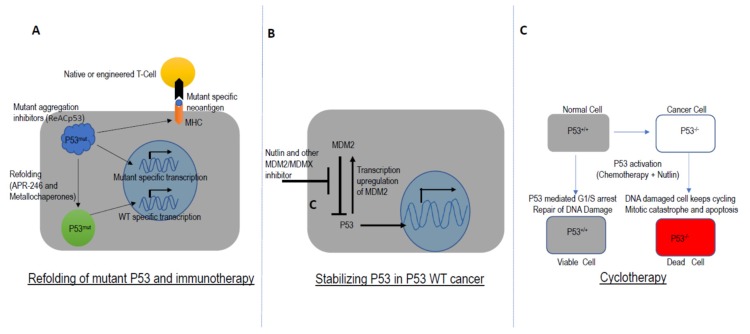
Targeting P53 in Drug Development. (**A**) APR-246 and similar compounds refold mutant P53 into wild-type-like conformation. Some P53 mutants are prone to aggregation and ReACp53 inhibits this aggregation. Mutant P53 can be presented on the surface of cancer cells as a neoantigen, providing an opportunity for the development of immunotherapy. (**B**) In P53 WT cancer cells, nutlin and other MDM2/MDMX inhibitors promote the accumulation and activity of WT P53. (**C**) Cyclotherapy protects normal cells from genotoxic agents. Nutlin arrests WT P53 containing normal cells, while P53 mutant cancer cells continue cycling and die due to accumulation of DNA damage.

Another promising approach has been the deployment of compounds which can restore the wild-type function of P53. Small molecules and peptides have been designed to stabilize P53 mutant proteins [119,120,121,122,123]. Metallochaperones have been reported to restore the function of mutant P53 by zinc incorporation [124]. Similarly, inhibition of amyloid-like structure formation in aggregation-prone mutants of P53 has shown promise as a therapeutic strategy in *TP53*-deficient tumors [124,125]. For example, APR-246 has been reported to reactivate mutant P53 and is currently in clinical development [119] (Figure 7A).

MDM2, and E3 ubiquitin ligase, regulates P53 activation in multiple ways including interaction with the transactivation domain of P53 and inhibition of P53 activity [126,127]. MDM2 also facilitates P53 nuclear export and can negatively regulate P53 through ubiquitin-mediated degradation by the proteasome [128,129,130,131]. In normal cells, MDM2 helps maintain low levels of P53 by inducing continuous ubiquitin-mediated degradation of P53. However, in response to cellular stress (eg, DNA damage, hypoxia, oncogenic activation), the interaction between MDM2 and P53 is disrupted, which leads to stabilization of P53. Interestingly, P53-mediated transcription also regulates MDM2. Thus, MDM2 and P53 are closely linked to each other through an autoregulatory negative feedback loop [132,133]. MDM2 is overexpressed in some plasma cell leukemia patients and several MM cell lines, resulting in inhibition of P53 activity [134]. Stabilization of P53 by inhibiting the MDM2-P53 interaction offered a novel strategy and led to development of Nutlin [116,135] (Figure 7B). Nutlin was reported as the first inhibitor of the P53-MDM2 interaction which demonstrated synergistic activity in MM with known anti-MM agents such as bortezomib, melphalan, and etoposide [135,136,137]. However, Nutlin is only effective in cells with wild-type P53, making it ineffective in del17p and mutant P53 backgrounds. Phase 1 trials with MDM2 antagonists in leukemia and liposarcoma have exhibited dose-limiting toxicities including neutropenia and thrombocytopenia [138]. 

The focus on MDM2 antagonism also led to the development of the concept of cyclotherapy (Figure 7C). These agents induce transient cell cycle arrest by stabilizing P53 in WT cells while P53 mutant cells continue to divide and exhibit enhanced sensitivity to chemotherapeutic agents [139]. The clinical development of cyclotherapy agents as well as preclinical efforts to discover cyclotherapeutic drug combinations are currently ongoing [140,141]. 

Another potential anticancer therapy in WT P53 containing tumors is based on cellular senescence, a known barrier to tumorigenesis [142]. Mouse cancer models have shown clear evidence of P53-dependent senescence in tumor suppression, and P53-induced senescence by MDM2 antagonists have shown promise as anticancer therapy [143,144,145,146,147,148]. 

Attempts to reintroduce wild-type P53 using gene therapy have unfortunately remained unsuccessful. Adenovirus-mediated transfer of wild-type P53 in ovarian cancer patients failed in randomized phase II/III trial [149]. It has also been shown that mere expression of wild-type P53 is not sufficient to arrest the growth of all transformed cells. 

P53-specific antigenic peptides can be presented on major histocompatibility complex molecules from tumor cells overexpressing mutant P53 and can evoke an antitumor immune response. Vaccination against mutant P53 has been shown to be effective in tumor-bearing mice [150,151]. Several peptide vaccines and dendritic cell vaccines utilizing mutant P53-targeted immunotherapy are in clinical development [152,153,154,155]. A number of Phase I/II immunization trials have been conducted so far using P53 immunogens, but unfortunately none of them have shown acceptable clinical efficacy [156]. 

## 6. Conclusion and Future Perspective

There are 3 distinct segments of patients with *TP53* dysregulation that have been identified in MM: monoallelic mutation, del17p, and biallelic inactivation. Based on analyses of the MGP data, the biallelic and high CCF del17p patients appear to have poorest prognosis, while the prognostic role of monoallelic mutations is less clear. Analysis of longitudinal patient samples is needed to more fully understand the timing and sequence of these aberrations as well as clinical outcomes following treatment with specific therapeutic regimens in each patient subset. Further, additional research is needed to identify co-occurring genetic interactions of P53/del17p dysregulation in MM. A reverse translational approach could identify dysregulated pathways and disease drivers in these segments from patient sample analysis. This approach could lead to discovery of new targets and eventually to new therapies to address the unmet medical need of these MM patients.

## Figures and Tables

**Table 1:** Del17p in NDMM Datasets ^1^.

Author	N	Prevalence Del17p in Full Dataset	Method	Range (Median) of % Positive Cells	Threshold/CCF for High-Risk	mPFS	mOS
Chang [56]	105	9.5% (*n* = 10)	FISH	18–95% *(Median 53%)*	None	Median 7.9 mo	Median 14.7 mo
Avet-Loiseau [43]	532	11% (*n* = 58)	FISH	32–94% *(Median 75%)*	≥60% PCs	mEFS 14.6 mo	Median 22.4 mo
Neben [51]	289	10% (*n* = 29)	FISH	NR	60–70% PCs	3-yr: 27%	3-yr: 50%
Lode [25]	92	57% *n* = 54)	FISH	NR	≥60% PCs	NR	NR
Boyd [50]	85	100% (*n* = 85) *(selected population of del17p patients)*	FISH	NR	None	14.7 mo	26.6 mo
An [49]	333	6.6% (*n* = 22)	FISH	25–100% *(Median 65%)*	>50% PCs	4 mo	16 mo
Lonial [52]	646	32% (*n* = 206)	FISH	NR	≥1 cell	NR	NR
Thanendrarajan [44]	779	10% (>20% cutoff; *n* = 76) 8% (>40% cutoff; *n* = 62) 7% (>60%; cutoff *n* = 51) 4% (>80% cutoff; *n* = 34)	FISH	Investigated >20, >40, >60, and >80% cutoffs	≥20% PCs	3 yr: 61%	3 yr: 67%
Shah [48]	1905	9% (*n* = 175)	MLPA	NR	NR	NR; HR: 1.57	NR; HR: 2.10
Shah [47]	1777	10.8% (MLPA < 0.8) (*n* = 192)	MLPA	Investigated > 0.8, 0.7–0.79, 0.55–0.69, and <0.55 cutoffs	MLPA value >0.8 (>20% PCs)	NR	≥0.7 to <0.8: HR = 1.8 ≥0.55 to <0.7: HR = 3.1 <0.5: HR = 2.2
Gaballa [57]	145	23.4% (*n* = 34)	FISH	NR	NR	8 mo	21 mo
Lakshman [58]	310	100% (*n* = 310) *(selected population of del17p patients)*	FISH	8–100% *(Median 69.5%)*	Investigated ≥ 20 vs. <20% ≥30 vs. <30% ≥40 vs. <40% ≥50 vs. <50% ≥60 vs. <60%	19.2 vs. 32.5 18.8 vs. 30.8 18.3 vs. 30.8 17.8 vs. 30.3 16.8 vs. 28.3	45.3 vs. NR 45.2 vs. 89.6 45.2 vs. 89.6 44.8 vs. 58.3 38.1 vs. 58.3
Thakurta [6]	605	100% (*n* = 605) *(selected population of del17p patients)*	FISH (discovery)	Investigated CCF range 0.3 to 0.8	>0.55 CCF	14.3 mo	36.1 mo
	235	100% (*n* = 235) *(selected population of del17p patients)*	FISH (replication)		>0.55 CCF	17 mo	32 mo
	108	100% *(selected population of del17p patients [n* = *108] from n* = *1273 MGP)*	NGS		>0.55 CCF	26 mo	36 mo

^1^ 3-yr = 3-year estimates; del17p = deletion of chromosome 17p; FISH = fluorescent in situ hybridization; HR = hazard ratio; PCs = plasma cells; CCF = cancer clonal fraction; IFM = Intergroupe Francophone de Myélome; MGP = Myeloma Genome Project; MLPA = multiplex ligation-dependent probe amplification; mo = month; mPFS = median progression free survival; mEFS = median event free survival; mOS = median overall survival; ND = not determined; NGS = next generation sequencing; NR = not reported; vs. = versus; yr = year.

**Table 2:** Del17p in RRMM Datasets ^1^.

Author	Total N	Prevalence Del17p in Full Dataset	Method	Range (Median) of % Positive Cells	Threshold/CCF for High-Risk	mPFS	mOS
Lakshman [39]	228 (152 control + 76 acquired del17p)	33% (*n* = 65)	FISH	9–100% *(Median 89%)*	None	23.0 mo (from diagnosis) 5.4 mo (after detection of del17p)	68.2 mo (from diagnosis) 18.1 mo (after detection of del17p)
Chin [59]	188	22.3% (*n* = 42)	FISH	NR	None	NR; Mixed NDMM and RRMM	NR
Chang [60]	85	22% (*n* = 17)	FISH	NR	>10%	*del17p+* vs. *neg.* 5.4 vs. 5.0 ns *p* = 0.60	11.5 vs. 15 ns P = 0.41
Chen [61]	88	15% (*n* = 13) 13% (*n* = 11)	FISH IHC	NR 10–90% (40%)	>10% >10%	*P53/del17p+* vs. *neg ^2^* 3.4 vs. 11 mo 3.4 vs. 11 mo	12.1 vs. 28.8 mo 7.2 vs. 28.8 mo
Avet-Loiseau [55]	552	10% (*n* = 69) Ixazomib Rd *n* = 36 placebo-Rd *n* = 33	FISH	Investigated >5% >20% >60%	None	*IRd* vs. *Rd* 21.4 vs. 9.7 21.4 vs. 6.7 15.7 vs. 5.1	NR

^1^ del17p = deletion of chromosome 17p; FISH = fluorescent in situ hybridization; HR = hazard ratio; CCF = cancer clonal fraction; neg = negative; NDMM = newly-diagnosed multiple myeloma; IHC: Immunohistochemistry; IRd = ixazomib + lenalidomide + dexamethasone; mo = month; mOS = median overall survival; mPFS = median progression free survival; neg = negative; NR = not reported; Rd = lenalidomide + dexamethasone; RRMM = relapsed/refractory multiple myeloma; vs. = versus. ^2^ Presence of nuclear P53 by IHC or del17p by FISH vs. patients who were negative for P53/del17p by the same method.

**Table 3:** In Silico Predicted Functional Consequence of *TP53* Mutations in MM ^1^.

	Mutation	Score and Predicted Impact		
		Mutation Assessor	SIFT	PolyPhen2
1	Y126C	3.25, Medium	0.00, Deleterious	1.00, Probably damaging
2	Y126H	3.25, Medium	0.00, Deleterious	1.00, Probably damaging
3	Y126splice	NA	NA	NA
4	S127F	3.29, Medium	0.00, Deleterious	1.00, Probably damaging
5	M133K	0.00, Neutral	0.00, Deleterious	0.12, Benign
6	M133T	0.00, Neutral	0.00, Deleterious	0.09, Benign
7	F134S	2.00, Medium	0.00, Deleterious	1.00, Probably damaging
8	F134L	2.64, Medium	0.00, Deleterious	1.00, Probably damaging
9	C135Y	3.08, Medium	0.00, Deleterious	1.00, Probably damaging
10	C135FS	NA	NA	NA
11	A161T	2.99, Medium	0.00, Deleterious	1.00, Probably damaging
12	Y163D	3.17, Medium	0.00, Deleterious	1.00, Probably damaging
13	Y163C	3.17, Medium	0.00, Deleterious	1.00, Probably damaging
14	R175G	3.28, Medium	0.00, Deleterious	1.00, Probably damaging
15	R175H	2.58, Medium	0.11, Tolerated	0.31, Benign
16	G199V	3.11, Medium	0.00, Deleterious	1.00, Probably damaging
17	Y234C	2.99, Medium	0.00, Deleterious	0.99, Probably damaging
18	R248W	3.28, Medium	0.00, Deleterious	1.00, Probably damaging
19	R248Q	2.94, Medium	0.00, Deleterious	1.00, Probably damaging
20	P250L	3.27, Medium	0.00, Deleterious	1.00, Probably damaging
21	R267W	3.22, Medium	0.05, Tolerated	0.73, Probably damaging
22	R273L	3.18, Medium	0.00, Deleterious	0.99, Probably damaging
23	R273H	2.08, Medium	0.13, Tolerated	0.63, Probably damaging
24	R282G	2.46, Medium	0.03, Deleterious	0.28, Benign
25	E285K	3.04, Medium	0.13, Tolerated	0.98, Probably damaging
26	R337C	1.56, Low	0.09, Tolerated	0.34, Benign
27	R337L	2.95, Medium	0.01, Deleterious	0.91, Probably damaging

^1^ Predicted functional consequences of mutations by three different in silico methods. Mutation Assessor classifies mutations based on functional impact score: ≤0.8 as neutral, 0.8–1.9 as low, 1.9–3.5 as medium and >3.5 as high. SIFT (Sorting Intolerant From Tolerant) classifies scores of <0.05 as deleterious. PolyPhen2 (Polymorphism Phenotyping) scores range from 0 (benign) to 1 (probably damaging).
